# Adolescents’ Characteristics and Peer Relationships in Class: A Population Study

**DOI:** 10.3390/ijerph19158907

**Published:** 2022-07-22

**Authors:** Elisa Cavicchiolo, Fabio Lucidi, Pierluigi Diotaiuti, Andrea Chirico, Federica Galli, Sara Manganelli, Monica D’Amico, Flavia Albarello, Laura Girelli, Mauro Cozzolino, Maurizio Sibilio, Arnaldo Zelli, Luca Mallia, Sara Germani, Tommaso Palombi, Dario Fegatelli, Marianna Liparoti, Laura Mandolesi, Fabio Alivernini

**Affiliations:** 1 Department of Human, Philosophical and Educational Sciences, University of Salerno, 84084 Fisciano, Italy; lgirelli@unisa.it (L.G.); mcozzolino@unisa.it (M.C.); msibilio@unisa.it (M.S.); 2 Department of Developmental and Social Psychology, Sapienza University of Rome, 00185 Rome, Italy; fabio.lucidi@uniroma1.it (F.L.); andrea.chirico@uniroma1.it (A.C.); monica.damico@uniroma1.it (M.D.); flavia.albarello@uniroma1.it (F.A.); sara.germani@uniroma1.it (S.G.); tommaso.palombi@uniroma1.it (T.P.); dario.fegatelli@gmail.com (D.F.); marianna.liparoti@gmail.com (M.L.); fabio.alivernini@uniroma1.it (F.A.); 3 Department of Human Sciences, Society and Health, University of Cassino and Southern Lazio, 03043 Cassino, Italy; p.diotaiuti@unicas.it; 4 Department of Movement, Human and Health Sciences, University of Rome “Foro Italico”, 00185 Rome, Italy; federica.galli@uniroma1.it (F.G.); arnaldo.zelli@uniroma4.it (A.Z.); luca.mallia@uniroma4.it (L.M.); 5 National Institute for the Evaluation of the Education System (INVALSI), 00153 Rome, Italy; sara.manganelli@invalsi.it; 6 Department of Humanities, University of Naples “Federico II”, 80133 Naples, Italy; laura.mandolesi@unina.it

**Keywords:** academic achievement, classmates, CSIQ, gender, immigrant background, latent variables, peer acceptance, peer friendship, social relationships with peers, socioeconomic status

## Abstract

Background: This study aimed to investigate differences in adolescents’ social relationships with classmates of diverse gender, socioeconomic status, immigrant background, and academic achievement. Methods: A population of 10th-grade students (N = 406,783; males = 50.3%; *M*_age_ = 15.57 years, *SD*_age_ = 0.75) completed the Classmates Social Isolation Questionnaire (CSIQ), an instrument specifically designed to measure two distinct but correlated types of peer relationships in class: peer acceptance and peer friendship. To obtain reliable comparisons across diverse adolescent characteristics, the measurement invariance of the CSIQ was established by means of CFAs and then latent mean differences tests were performed. Results: Immigrant background, academic achievement, and socioeconomic status all proved to be important factors influencing relationships with classmates, while being a male or a female was less relevant. Being a first-generation immigrant adolescent appears to be the foremost risk factor for being less accepted by classmates, while having a low academic achievement is the greatest hindrance for having friends in the group of classmates, a finding that diverges from previous studies. Conclusions: This population study suggests that adolescent characteristics (especially immigrant background, socioeconomic status, and academic achievement) seem to affect social relationships with classmates.

## 1. Introduction

Social relationships with peers have a crucial role among adolescents [1,2], who increasingly tend to seek emotional support outside the family [3,4,5,6], and to establish more intimate social interactions with their peers [7,8]. Peer social relationships are exceptional, as “they are more equal, less controlling, and less judgmental than relationships with parents and other adults” [2] (p. 541). They are beneficial for developmental outcomes [9,10,11,12] and they sustain increased autonomy [7] and the development of identity [13,14]. Nevertheless, they are also highly complex and nuanced [15,16].

Not all peers are equal: Adolescents spend most of their daily life at school where they experience both positive and negative social interactions [17,18] and, even when they are not physically there, they tend to think about what has happened, or might happen, at school [6,19]. Although classmates do not choose each other, they interact on a daily basis, also by posting items, messaging or chatting on their mobile phones. In fact, easy access to modern communication technology has shaped the way adolescents develop and sustain their mutual interactions [20,21]. Classmates share their time and schoolteachers, as well as most of their educational and life experiences, usually within the same physical spaces. Several studies have shown the importance of creating flexible and technological learning environments to sustain students’ engagement and interaction among their peers [22,23,24] and to counteract social isolation [25]. The group of classmates is therefore one of the most important socialization and reference groups for adolescents [26], as it is their main social arena for coordinating school activities, in which they share space and time, also outside school.

### 1.1. Different Kinds of Social Relationships with Peers in Class

In investigating classmates’ social relationships, it is important to be aware that there are various different types [18,27,28,29]. For example, studies conducted in the class context have identified two related, but conceptually distinct aspects of peer social relationships, i.e., peer acceptance and peer friendship [15,27,28,30,31,32,33]. Peer acceptance pertains to the individual’s perceived level of inclusion within the group. It can be measured by the number of social interactions with classmates at school [34], including casual social interaction and general contact with others in the classroom, such as simply talking to them [17,34]. An extensive body of research has indicated that higher levels of peer acceptance are related to more adaptive social behaviors [35,36], school adjustment [29,37,38,39,40,41] and psychological well-being [30].

Although classmates often share the same space, only some of them become friends [18]. Friendship entails a more intimate and meaningful connection. It is a voluntary mutual relationship [42,43,44], involving social support [45], the sharing of secrets [46] and shared leisure activities [47]. Friendships are characterized by individual similarities such as interests in common [1], and, for adolescents, it implies frequent contact and sharing a great deal of spare time without parental control, also outside school [1,48,49,50,51]. Previous research has shown that having close friendships is important for academic attainment [52,53,54], higher self-esteem [55,56], psychological well-being [30,57], and healthy emotional functioning [58,59,60]. In a recent meta-analysis based on 22 studies, Wentzel and colleagues [61] showed that having friends at school is related to academic benefits, both in terms of cognitive as well as performance outcomes.

Although these two types of relationships are particularly relevant and can shape adolescents’ interactions with the social environment [62], there is some evidence to suggest that not all adolescents benefit equally from the positive influence of peer acceptance and peer friendship [30,63,64].

### 1.2. Adolescents with Different Characteristics and Their Social Relationships with Peers in Class

#### 1.2.1. Male or Female Adolescents

Being male or female has long been seen as an important variable when considering relationships with peers. Girls are considered to be more oriented toward other peers [65], they show higher-quality relationships than males [66], better peer communication [67] and their relationships are often more characterized by prosocial behaviour [68,69]. Other studies have, however, suggested that they are less socially connected with their classmates than boys [30]. In addition, they claim to have more stressful events in their relationships with peers [29,65,68], and they appear to be more vulnerable when their peer connections end [70]. Male peer relationships seem to be characterized by a larger size of the group and a greater number of connections [6]. Boys also report higher levels of perceived popularity than girls [71], while also having higher levels of negative interactions with their peers, for example, they are more likely than girls to be victims of overt bullying behaviors [72,73]. The influence of gender on peer relationships in class appears to be particularly complex, and it is still unclear which group is more at risk of being socially isolated [68,74]. More conclusive data would be important for supporting the more vulnerable group in developing and maintaining peer relationships, which in turn could facilitate positive adjustment at school [75]. We have made no explicit hypothesis in the present study, since the results of previous research have proved to be controversial.

#### 1.2.2. Adolescents with Low or High Socioeconomic Status (SES)

Adolescents from low-SES families appear to have less social contacts [16,76,77,78], fewer friends [79,80,81] and lower levels of perceived popularity [82] than their peers from higher SES backgrounds. In addition, they appear to be less accepted and more frequently rejected by peers [83], less socio-emotionally competent [84], more vulnerable to social isolation [16,81], and more subject to adverse peer relationships [85]. The evidence reviewed so far, therefore, suggests that having a less affluent family influences peer relationships in a negative way. However, further investigation is needed to understand if SES has different effects on peer acceptance and peer friendship. Some studies have found that adolescents from lower SES families have fewer friends at school, and tend to be less accepted by their classmates [30]. Other studies conducted on adolescents who live in poverty indicate that they particularly benefit from friendships while peer acceptance appears to be ineffective for their school adjustment [35]. In the present study, we expected that adolescents from more affluent families would have more peer relationships. Conversely, we made no explicit predictions related to the differences between peer acceptance and peer friendship due to belonging to either a low or a high SES family, since there are still very few studies which have clearly distinguished between these two factors.

#### 1.2.3. Native Adolescents or Adolescents with an Immigrant Background

It seems that peer acceptance and peer friendship play a pivotal role for immigrant adolescents in fostering processes of acculturation and social integration, which mainly occur in the school context [86,87], and can be facilitated by using communication technologies as intercultural connectivity tools [88,89]. Previous studies conducted in primary schools have shown that immigrant pupils have less peer relationships and a greater degree of victimization and exclusion than their native-born peers [30,31,35,90,91,92,93]. This appears to be the case also for older immigrant adolescents, who reported having fewer friends than native-born adolescents [94], and to be more socially excluded in general [95]. This is unfortunate because previous studies suggested that peers can play an important role in the positive adaptation of young immigrants [95,96,97].

The time spent in the country of immigration also seems relevant for the inclusion process [98]. From an acculturation perspective [95], it is to be expected that second-generation immigrants would be less socially excluded than those of the first generation, as they have spent more time in the host country, and have therefore had more opportunities to establish close relationships with their peers. The literature has mostly confirmed this hypothesis, indicating that second-generation adolescents are less excluded [95], have more friends [99], and experience less antisocial behaviors than their first-generation peers [30,95], while first-generation adolescents have an increased risk of loneliness [100]. In the present study, we expected that adolescents with an immigrant background would report fewer social connections compared to their native peers, and that second-generation immigrant adolescents would have more social relationships with classmates than their first-generation peers. We also set out to investigate the possibility of variations in these results depending on the specific type of peer relationship considered (i.e., peer acceptance and peer friendship), bearing in mind that a recent study [30] has shown that there was no difference in peer acceptance between first- and second-generation immigrant adolescents, whereas the latter tended to have more friends. These results seem to indicate that friendship, rather than acceptance, benefits from the length of time spent in the country of immigration.

#### 1.2.4. Adolescents with Low or High Academic Achievement

Among all possible peer groups with whom an adolescent interacts, and that can influence academic achievement, that of classmates has been shown to have the most robust effect as “students do not learn alone but rather in the presence of many peers” [101] (p. 438). Most educational and academic activities take place in the context of this group [17,102,103,104], and there is a large body of research on the benefits of peer relationships on academic achievement (recently, [105,106,107,108]). Unfortunately, very few studies have investigated the opposite effect, i.e., that of adolescents’ academic achievement on their social relationships. Academic achievement is an important indicator of an adolescent’s ability to adapt to the school environment [64,109,110], and several studies have demonstrated that there is a relationship between high levels of academic achievement and self-regulation [111], positive emotions [112,113,114,115,116,117] or social inclusion [64]. It is therefore to be expected that students with higher academic results might have more peer connections within the context of the class. To the best of our knowledge, ours is the first study to focus specifically on the role of academic achievement in shaping classmates’ peer relationships during adolescence. As regards the possible differences between peer acceptance and peer friendship, previous studies have shown that the degree of social inclusion in a peer group can be affected by students’ academic skills [118,119,120], which are often highly valued. On these premises in the present study we expected that academic achievement would be more important for peer acceptance rather than for peer friendship.

### 1.3. Obstacles to Establishing Meaningful Differences in Classmates’ Relationships

Since peer acceptance and peer friendship cannot be observed directly, they need to be inferred from observable indicators such as responses to the items of a questionnaire specifically designed to reflect these latent variables. When attempting to establish differences in a certain latent variable across two or more groups [121], as in the present study, the typical procedure in the social sciences is to compare the scale scores obtained by adding or averaging out the item scores, and considering the observed means as being equal to the means of the latent variables [122]. However, in this practice, the potential non-equivalence of the measurement across different groups can constitute an important source of bias [123]. The items of the various scales might be interpreted differently by adolescents with different individual characteristics. For example, during adolescence girls tend to develop closer friendships and to rely more on friends [29], which could lead them to interpret items pertaining to social relationships in more intimate terms than boys would do. In order to make meaningful comparisons, the invariance of measures across groups must therefore be proven. In the present study, we adopted a well-established procedure based on a series of sequential steps [123,124,125,126]. First, we established configural invariance which means that adolescents with different characteristics (for example, boys and girls, or natives and immigrant adolescents) similarly conceptualize the structure of peer relationships as two distinct concepts (i.e., peer acceptance and peer friendship), which fully correspond to their respective items (latent variables) [30,127]. When this level of invariance has been established, the second step is to provide evidence for the metric invariance, which means that adolescents with different characteristics interpret items about peer relationships in the same way. If metric invariance is present, the association between the latent variables referring to peer relationships (i.e., peer acceptance and peer friendship) and the corresponding items is equal across adolescents with different characteristics. A third step is to ascertain scalar invariance, i.e., that adolescents with the same type and level of peer relationships have chosen the same response options for the same items, regardless of their individual characteristics. When scalar invariance exists, the latent means can be meaningfully compared across the groups [127].

Another source of potential bias is related to sample size. In social sciences, an adequate sample size is a priority when designing a study [128,129] as larger samples permit more precise parameter estimates while increasing the statistical power of the study [130,131,132]. Unfortunately, in empirical research it is not always possible to obtain very large samples due to constraints regarding time, funding, or recruitment. Our study is based on a population and it necessarily includes all the possible samples that we wanted to draw conclusions about, so it allowed us to obtain all the precise parameters of interest. A population eliminates sampling error and gives an accurate picture of the heterogeneity between individuals. Therefore, we can safely say that the differences we observed in classmates’ acceptance and friendship across different groups of adolescents correspond to the genuine differences existing in a population of 10th-grade students. Another benefit of larger samples is the possibility of evaluating small effects in the data that might otherwise have been undetected, and this is particularly the case for a population. This advantage is counterbalanced by the fact that these distinctions might be so small as to be trivial, so it is important to consider their effect size [133]. While *p*-values inform us about the presence or absence of an effect, the effect size quantifies its magnitude [134]. In the present study, we used Cohen’s *d* as a standardized measure of the effect size, and we evaluated the differences between the groups in our data, in terms of trivial or non-trivial effects.

### 1.4. The Present Study

The present study aims to investigate the differences in peer acceptance and peer friendship across adolescents with different characteristics (gender, SES, immigrant background, and academic achievement). The research questions addressed by the present study can be summarized as follows:

- Do peer acceptance and peer friendship differ across male and female adolescents?
- Do peer acceptance and peer friendship differ across adolescents with varying socioeconomic backgrounds (low, middle, and high)?
- Do peer acceptance and peer friendship vary across adolescents with different immigrant backgrounds (native students and first- and second-generation immigrants)?
- Do peer acceptance and peer friendship vary across adolescents with different levels of academic achievement (low, average, and high)?

## 2. Materials and Methods

### 2.1. Sample and Procedure

The data analyzed in the present study are based on the population of 406,783 Italian 10th-grade students (*M*_age_ = 15.57 years; *SD*_age_ = 0.75; N_classes_ = 24,102) who took part in the paper and pencil National Evaluation of Learning in 2014 (https://www.invalsi.it/invalsi/index.php, accessed on 7 June 2018). The National assessment included standardized tests elaborated by the Italian National Institute for the Evaluation of the Education System (INVALSI) that covered reading comprehension in Italian, reasoning and mathematical skills. In addition, it included a Student Questionnaire that contained the CSIQ as well as demographic questions. These instruments were administered in class during ordinary school days.

The data that corroborates the findings of the present study are available at: https://invalsi-serviziostatistico.cineca.it (accessed on 7 June 2018). It is important to acknowledge that, although the present study analyzed data from a population of students, they were cross-sectional and limited to one specific school grade (10th grade).

The survey was conducted according to the ethical guidelines of the INVALSI [135] which also reviewed and approved it. To ensure conformity with ethical issues, each school provided parental permission and informed consent, according to the national assessment protocol [136]. All of the participants were given a standardized introduction to the survey, which informed them of its purpose and gave them instructions on how to complete the questionnaire.

### 2.2. Measures

#### 2.2.1. The Classmates Social Isolation Questionnaire (CSIQ)

The CSIQ is a short and concise specific measure focused on peer relationships exclusively with classmates. This choice was made to emphasize the crucial role of classmates as a socialization group. The measure includes various types of social contact, ranging from fairly superficial connections to close friendships, and it focuses exclusively on the number of social contacts with peers [90].

The CSIQ consists of 8 items which tap the domains of peer acceptance and peer friendship: four items were used for the former (e.g., “How many of your classmates speak with you?”) and four for the latter (e.g., “How many of your classmates do you meet outside school?”). For each of the 8 items, students were asked to indicate the number of their classmates with whom they have social relationships on a 5-point scale (None, Few, Some, Many, All). The lowest possible score denotes no social connections between the respondent and any of their classmates, while the highest possible score indicates that they report social contact with everyone in the class. Therefore the scale assumed that there is a single bipolar continuum running from the absence of social contacts at one end to a high degree of social connections at the other.

Previous studies [90,137] have provided initial evidence about the psychometric properties of the CSIQ: the bidimensional structure of the CSIQ has been assessed by means of explanatory and confirmatory factor analyses and the scale has proved to be invariant across children with diverse gender and immigrant background (first- and second-generation). Moreover, the criterion validity of the CSIQ has been supported. In the present study, the coefficient alpha for peer acceptance was 0.77, and for peer friendship it was 0.83. In Appendix A the English and Italian versions of the CSIQ are presented, as well as the scoring procedure.

#### 2.2.2. Adolescents’ Characteristics

##### Male or Female Adolescents

Gender was coded into two categories, with 1 indicating males and 2 indicating females.

##### Adolescents with Low or High SES

Adolescents’ socioeconomic status (SES; Organization for Economic Co-operation and Development (OECD) [138]) was measured by calculating the factor scores deriving from a Principal Component Analysis (PCA) on four indicators: (1) occupational level of parents, (2) educational level of parents, (3) home possessions, and (4) home literacy resources. The tertiles of the SES scores were then computed in order to distinguish three groups of students: lower SES (SES scores in the first tertile), medium SES (SES scores in the second tertile), and higher SES (SES scores in the third tertile).

##### Native Adolescents or Adolescents with an Immigrant Background

Immigrant background was defined in accordance with the classification of OECD [138]: native adolescents were defined as born in Italy and with at least one parent who was born in Italy; first-generation immigrants were defined as foreign-born and with parents born abroad; second-generation immigrants were defined as born in Italy and with parents born abroad. In the present study immigrant background was coded by means of two dummy variables (0/1), one for the first generation and one for the second generation, with native adolescents as the reference category.

##### Adolescents with Low or High Academic Achievement

Academic achievement was measured through the mark that students had obtained in the national state examination at the end of the first cycle of education in Italy (grade 8) and it is a requirement to access the second cycle of education (high school). The final pass mark for the state examination ranges from 6 to 10 with honors. In the present study it was expressed as a whole number from 6 to 11 (with 11 indicating 10 with honors) and then it was coded into three categories, with 1 indicating a mark equal to 6, with 2 indicating a mark equal to 7 or 8 and 3 a mark greater than 8. The first category includes students who obtained the minimum level of achievement to access the second cycle of education. The second group includes students who, according to the Italian Ministry of Education, have reached an intermediate level of achievement [139], while the third category included high achiever students [139].

#### 2.2.3. Analysis

The analyses were carried out using Mplus 8 [140]. In order to take into consideration the hierarchical structure of our data (i.e., students are nested within classes), the “Type = complex” analytic approach and the Maximum Likelihood estimation with robust standard errors (MLR) were used. To examine the psychometric properties of the CSIQ, confirmatory factor analyses (CFAs) were conducted on the population of 10th grade students and the goodness-of-fit of the models with the data was assessed using the chi-square test statistic and alternative fit indices (comparative fit index (CFI), root mean square error of approximation (RMSEA), and standardized root mean square residual (SRMR)), according to the cut-off values for well-fitting models [141,142]. In line with our theoretical expectations, we tested a model consisting of two correlated, but distinct factors (peer acceptance and peer friendship) [31,90]. This two-factor model was then compared to a more parsimonious model with just one factor. Subsequently, we examined the measurement invariance of the scales across adolescents with different characteristics, i.e., being male or female, having a low-SES, middle SES or high-SES family, having or not having an immigrant background (first-and second-generation) and being a low, average or high achiever, by means of a hierarchical series of multigroup confirmatory factor analyses, imposing increasingly restrictive equality constraints on the model’s parameters [143]. In each step of the analysis, the fit of the nested models was compared using the change in CFI values (∆CFI ≤ 0.01), which may be less sensitive to the sample size according to [144]. In these tests, the variances of the groups were constrained to be equal to 1, so that the results could be interpreted in terms of Cohen’s *d,* thus allowing comparisons across groups. The very small amount of missing data (ranging from 0.92% and 1.12%) was handled using the Full Information Maximum Likelihood method as implemented in Mplus [140].

## 3. Results

Descriptive statistics are shown in Table 1. Assumptions of normality were verified, ensuring that skewness and kurtosis were within acceptable ranges (±2; [129]; see Table 1).

**Figure 1:**
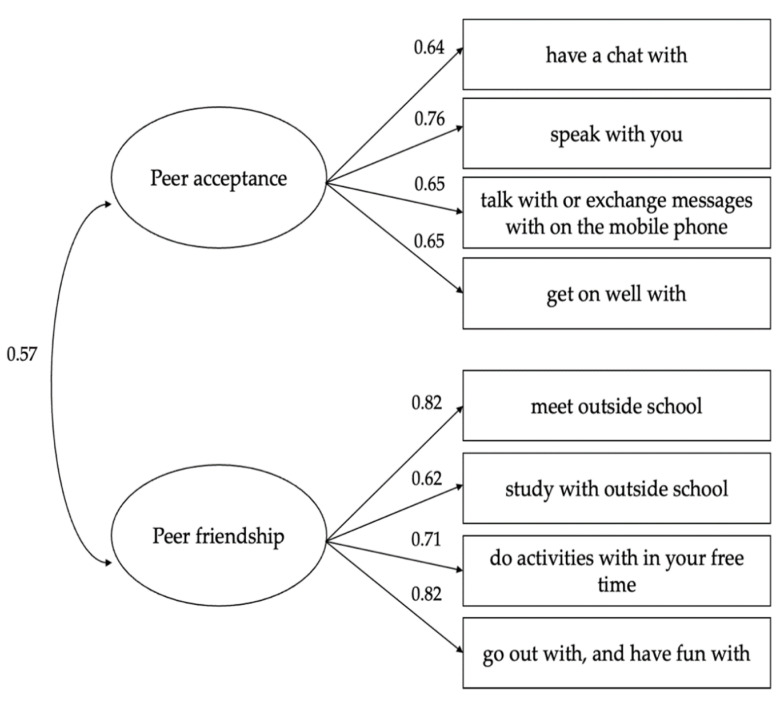
Confirmatory factor analysis results. Note: All of the values are standardized and are statistically significant at *p* < 0.001. In the model, measurement errors are not allowed to be correlated.

Except for the chi-square test (in the present study probably affected by the sample size), all the alternative fit indices for the model with two distinct dimensions indicated a good fit with the empirical data: χ^2^(19) = 35,936.248, *p* < 0.001; CFI = 0.957; RMSEA = 0.068 (90% confidence interval [CI] = [0.068, 0.069]); SRMR = 0.045. The standardized factor loadings ranged from 0.62 to 0.82, as shown in Figure 1. We also tested a solution with only one factor, but it had poor fit χ^2^(20) = 195,128.071, *p* < 0.001; CFI = 0.769; RMSEA = 0.155 (90% confidence interval [CI] = [0.154, 0.155]); SRMR = 0.097.

### 3.1. Measurement Invariance across Adolescents with Different Characteristics

The results of the multigroup CFAs across adolescents with diverse characteristics are presented in Table 2. The results of the comparison of the configural invariance model with the model in which all the factor loadings are constrained to be equal across groups confirmed the full metric invariance of the CSIQ across all the groups considered (for gender, ΔCFI = 0.002; for SES, ΔCFI = 0.004; for immigrant background, ΔCFI = 0.002; for achievement, ΔCFI = 0.003). The metric invariance models were compared to the models in which all the intercepts were constrained to be equal across the groups. The results provided support for the full scalar invariance of the scales across SES (ΔCFI = 0.004), immigrant background (ΔCFI = 0.002), and achievement (ΔCFI = 0.005) and partial invariance across gender (ΔCFI = 0.004). Standards for partial invariance commonly suggest that a factor can be considered invariant if the majority of items on the factor are invariant [126]. In our case the equality constraint of intercepts as regards gender was released just for Item 5 (“How many of your classmates do you talk with or exchange messages with on the mobile phone?”). Therefore, we can expect that partial invariance matters little in true mean differences.

### 3.2. Differences in Peer Acceptance and Peer Friendship across Adolescents with Diverse Characteristics

Considering the satisfactory level of measurement invariance, the differences in latent means between the various groups for peer acceptance and peer friendship were tested (Table 3). For each comparison, the latent factor mean was set to 0 in one group (the reference group) and allowed to vary in the other group [123]. For example, high achievers are 0.36 standard deviation higher than low achievers in their latent mean of peer acceptance and 0.47 standard deviation higher compared to low achievers in their latent mean of peer friendship.

#### 3.2.1. Male or Female Adolescents

Boys showed slightly higher levels of peer acceptance and peer friendship than girls, with a stronger impact on peer friendship than on peer acceptance.

#### 3.2.2. Adolescents with Low or High SES

Adolescents from high-SES families showed higher levels of social connections, both in terms of peer acceptance and peer friendship, compared to those with lower SES. The same pattern is present also for middle SES adolescents compared to those with low SES. This difference appears to be more marked in peer friendship than in peer acceptance.

#### 3.2.3. Native Adolescents or Adolescents with an Immigrant Background

Both first- and second-generation immigrant adolescents showed lower levels of peer acceptance and peer friendship than the native adolescents did. Moreover, first-generation adolescents had lower levels of peer acceptance and peer friendship than second-generation adolescents. As regards possible differences in peer acceptance and peer friendship, having an immigrant background appeared to be particularly relevant for being accepted in the classmates’ group and this is true for both first-and second-generation immigrants.

#### 3.2.4. Adolescents with Low or High Academic Achievement

Students who received average and high marks in the national state examination showed higher levels of both peer acceptance and peer friendship than the students who obtained a low final evaluation. This difference seems to be more evident in peer friendship than in peer acceptance.

### 3.3. Peer Relationships in Class: What Adolescents’ Characteristics Make a (Non-Trivial) Difference?

In Table 4, the adolescents’ characteristics are ordered according to their contribution as risk factors for peer acceptance and friendship in class (expressed in terms of Cohen’s *d*). The characteristics of adolescents with a larger effect size are therefore associated with less acceptance and fewer friendships within the class. In Table 4, only non-trivial effects (Cohen’s *d* > 0.19) are shown, in accordance with the most commonly used criteria [133].

Adolescents who self-reported that they were less accepted in class are: (1) those who have a first-generation immigrant background (compared to natives), followed by (2) low achievers (compared to high achievers), (3) adolescents who have a second-generation immigrant background (compared to natives) and (4) those who come from a lower-SES family (compared to those who come from a higher-SES family). All these differences have an effect size greater than 0.30. Adolescents who reported having fewer friendships in class are (1) those who obtain poor academic marks at school (compared to high achievers), (2) adolescents who come from less affluent families (compared to higher SES families) and (3) those who have a first-generation immigrant background (compared to natives). For having fewer friendships also, the effect size of all these differences is greater than 0.30. Average achievers (compared to high ones) and second-generation immigrant adolescents are also characterized by non-trivial (but less strong) differences as regards having fewer friendships in class (Cohen’s *d* > 0.19).

It is worth noting that some differences across adolescents’ characteristics amounted to less than 0.2 standard deviations (and these negligible differences are therefore not shown in Table 4), such as being a male (compared to being a female) and having a second-generation immigrant background (compared to being a first-generation immigrant).

## 4. Discussion

Adolescents’ characteristics have an influence on their relationships with classmates and the present study analyzed the specific contribution of each of these factors on the basis of a population of Italian 10th-grade students.

First, we established the psychometric properties and measurement invariance of the CSIQ, a time-efficient instrument specifically designed to assess relationships between classmates. The results showed that the posited model with two distinct but correlated factors (i.e., peer acceptance and peer friendship) fitted the data well. This is in line with previous studies conducted using the instrument on different populations [31,35,90]. The CSIQ also showed full configural and metric invariance across gender, SES, immigrant background, and academic achievement, meaning that adolescents with different characteristics appear to conceptualize their relationships with classmates and interpret the corresponding items in a very similar way. The full scalar invariance across SES, immigrant background, and academic achievement as well as the partial scalar invariance across gender was also confirmed. This allowed us to ascertain meaningful comparisons in peer acceptance and peer friendship across adolescents with different characteristics while taking measurement errors into account.

### 4.1. Differences between Male and Female Adolescents

As regards differences between boys and girls, previous studies have had mixed or contradictory results as to which group is more at risk of being socially isolated [68,74]. Our findings showed that males had slightly higher levels of peer relationships with their classmates than females, especially as regards peer friendship. However, gender seemed to play only a minor role in the context of social relationships with classmates, as indicated by its insubstantial effect size.

### 4.2. Differences between Adolescents with Low and High SES

In accordance with previous studies, we expected adolescents with a low-SES background to be less included and to have fewer friends than adolescents with a higher SES [16,30,79,80,83]. Our results confirmed this prediction and provided some new information, i.e., that this difference appears to be more marked in peer friendship than in peer acceptance. Leisure activities are important to the quality of life [145,146], and adolescents also spend a lot of time in each other’s company outside school. However, participation in social life outside school is often influenced by the economic ability to access social activities [16,81]. Adolescents from lower SES families therefore encounter many obstacles to accessing leisure time activities: for example, the cost of joining groups, transport expenses, the cost of material goods (e.g., clothes or smartphones), or the shame in asking their parents for money [79,80]. Moreover, adolescents’ economic resources are mainly provided by their parents, and they have few opportunities to change their economic situation [80]. Coming from a more affluent families thus appears to enable and encourage social relations with peers, especially outside the school context.

### 4.3. Differences between Native Adolescents and Adolescents with an Immigrant Background

It is well known that immigrant adolescents have a greater risk of being more socially excluded [95] and of having fewer friends [94] than their native peers. Our findings have confirmed this data and expanded it by showing that immigrants’ relationships with classmates have some peculiarities, depending on whether peer acceptance or friendship is considered. Having an immigrant background appears to have a negative influence above all on peer acceptance. During adolescence, there is a developmental pressure to conform to group norms in order to be accepted by peers [147], and it could be that adolescents with an immigrant background are under more pressure, as they are involved in both acculturative and developmental processes at the same time [148]. Despite the increasing number of people with an immigrant background, and all the efforts made to promote inclusive strategies at school, the social inclusion of pupils with an immigrant background is still a difficult issue [31,149] and the results of the present study have confirmed this fact in a population of 10th-grade students. This is unfortunate because, although acceptance is a less intimate and meaningful bond than friendship, casual forms of contact are more frequent and they can lead to new friendships which offer opportunities for intergroup contacts [150].

In accordance with an acculturation perspective [151], we expected that second-generation immigrants would be more accepted and would have more friends than first-generation adolescents, as they have an advantage in terms of cultural and socioeconomic stability by being born in the host country [151]. Our results showed that second-generation adolescents have slightly higher levels of relationships with classmates than first-generation immigrants, especially as regards peer friendship, but that the differences from one generation to the next does not appear to be particularly relevant. This finding sheds light on the importance of promoting an inclusive educational climate which encourages relationships between classmates, regardless of the generation of immigration [152].

### 4.4. Differences between Low or High Achievers

Our results showed that adolescents with better academic results have higher levels of both peer acceptance and peer friendship compared to those who are less academically successful. This result contrasts with previous research, according to which students who receive good grades at school are more likely to be socially excluded by their classmates, and to have fewer friends [153,154]. For example, some studies have shown that students whose classmates call them “brain”, “nerd” or similar terms tend to be more rejected by peers, and that they have increased levels of anxiety and loneliness [154,155,156]. Our findings indicate a positive influence of high academic achievement on social relationships with classmates, instead. Contrary to our expectations, this benefit was more marked for peer friendship than for peer acceptance. One possible explanation is that instrumental aid plays a role in this type of relationship. Instrumental aid is the giving of assistance, such as helping others with homework [157]. The utility of a friendship involves providing benefits for one’s friends [158] and it has been defined as “the degree to which relationships serve as a means to each friend’s desired ends” [158] (p. 261). From this point of view, it makes sense that adolescents with higher grades should have more friends because they can provide instrumental benefits to their classmates, in terms of academic success.

Some limitations of the present study should be mentioned. First, although our study analyzed the data from an entire population of students, it focused on one specific grade (10th grade). Future studies will therefore be needed to replicate these findings in other grades of higher secondary school. Secondly, it is important to acknowledge that the present study was based on cross-sectional data, whereas future research may benefit from a longitudinal investigation of the possible trajectories of social relationships between classmates during adolescence. This would, for example, make it possible to investigate interactions between peer acceptance and peer friendship over time.

## 5. Conclusions

In conclusion, the present study provides strong evidence for the importance of adolescents’ immigrant background, academic achievement, and SES in shaping their social relationships with classmates. Notably, being a first-generation immigrant adolescent appears to be the foremost risk factor for being less accepted by classmates, while having a low academic achievement is the greatest hindrance for having friends in the group of classmates. Classrooms are one of the most important social contexts where adolescents can explore their potential outside the confines of their family and where they can create and maintain interactions with their peers. It is therefore crucial for schools to focus not only on adolescents’ academic achievement but also on their social well-being at school.

## Figures and Tables

**Table 1:** Descriptive statistics for the study variables.

		Mean	*SD*	%	Skewness	Kurtosis
**Adolescents’** **characteristics**	Males	-	-	50.3%	-	-
	SES	0.30	0.99	-	−0.17	−0.42
	First-generation immigrant BKGD	-	-	5.0%	-	-
	Second-generation immigrant BKGD	-	-	3.6%	-	-
	Academic achievement	7.78	1.27	-	0.49	−0.29
**Peer acceptance**	Item 1	4.41	0.93	-	−1.62	1.96
	Item 3	4.13	0.98	-	−0.87	−0.12
	Item 5	3.61	1.02	-	−0.35	−0.61
	Item 7	3.70	1.04	-	−0.43	−0.68
**Peer friendship**	Item 2	2.71	0.94	-	0.29	−0.41
	Item 4	2.05	0.92	-	0.85	0.67
	Item 6	2.31	1.02	-	0.54	−0.28
	Item 8	2.60	1.00	-	0.35	−0.46

Note: *SD*, standard deviation; SES, socioeconomic status; BKGD, background.

**Table 2:** Goodness-of-fit indices for invariance of the CSIQ across adolescents with different characteristics.

	χ^2^	*df*	χ^2^/*df*	CFI	RMSEA	SRMR	Models Compared	ΔCFI
**Male/female adolescents**								
Configural model	37,365.157	38	983.294	0.956	0.070	0.045		-
Metric model	39,410.742	46	856.755	0.954	0.065	0.054	Metric against configural	0.002
Scalar model	50,699.543	52	974.991	0.941	0.069	0.057	Scalar against metric	0.013
Partial scalar model ^a^	42,532.752	51	833.976	0.950	0.064	0.055	Partial Scalar against Metric	0.004
**Adolescents with low/middle/high SES**								
Configural model	33,980.864	57	596.156	0.956	0.069	0.044		-
Metric model	36,573.236	73	501.003	0.952	0.063	0.061	Metric against configural	0.004
Scalar model	39,620.915	85	466.128	0.948	0.061	0.062	Scalar against metric	0.004
**Native adolescents/first-/second-generation immigrant adolescents**								
Configural model	33,470.570	57	587.203	0.956	0.068	0.044		
Metric model	34,936.501	73	478.582	0.954	0.061	0.051	Metric against configural	0.002
Scalar model	37,020.586	85	435.536	0.952	0.058	0.052	Scalar against metric	0.002
**Adolescents with low/average/high academic achievement**								
Configural model	35,712.609	57	626.537	0.956	0.068	0.045		-
Metric model	37,581.526	73	514.815	0.953	0.062	0.055	Metric against configural	0.003
Scalar model	41,819.627	85	491.996	0.948	0.061	0.055	Scalar against metric	0.005

Note: χ^2^, chi-squared; *df*, degrees of freedom; χ^2^/*df*, normative chi-square; CFI, comparative fit index; RMSEA, root mean square error of approximation; SRMR, standardized root mean square residual. ^a^ Equality constraint of intercepts was released for Item 5.

**Table 3:** Results of the latent mean differences tests.

Adolescents’ Characteristics		Peer Acceptance Mean Differences	Peer Friendship Mean Differences
**Gender**	Males (compared to females)	0.07	0.13
**SES**	High SES adolescents (compared to low SES adolescents)	0.31	0.38
	Middle SES adolescents (compared to low SES adolescents)	0.20	0.19
	High SES adolescents (compared to middle SES adolescents)	0.11	0.19
**Immigrant background**	First-generation immigrants (compared to natives)	−0.43	−0.37
	Second-generation immigrants (compared to natives)	−0.34	−0.20
	Second-generation immigrants (compared to first-generation immigrants)	0.10	0.17
**Academic achievement**	High achievers (compared to low achievers)	0.36	0.47
	Average achievers (compared to low achievers)	0.21	0.24
	High achievers (compared to average achievers)	0.15	0.24

Note: SES, socioeconomic status. For all comparisons, the latent factor variance is set to 1.0. All the values are statistically significant at *p* < 0.001. The results could be interpreted in terms of Cohen’s *d*.

**Table 4:** Adolescents’ characteristics and social relationships in class (ranking by effect size).

Adolescents Who Are Less Accepted in Class	Size of the Difference ^1^	Adolescents Who Have Fewer Friendships in Class	Size of the Difference ^1^
First-generation immigrants ^2^	0.43	Low achievers ^3^	0.47
Low achievers ^3^	0.36	Low SES adolescents ^4^	0.38
Second-generation immigrants ^2^	0.34	First-generation immigrants ^2^	0.37
Low SES adolescents ^4^	0.31	Average achievers ^3^	0.24
		Second-generation immigrants ^2^	0.20

Note: Only the differences with Cohen’s *d* > 19 are listed. Some comparisons are not displayed in Table 4: for peer acceptance, low achievers compared to average achievers = 0.21; low SES adolescents compared to middle SES adolescents = 0.20; for peer friendship, low achievers compared to average achievers = 0.24. ^1^ effect size: Cohen’s *d*; ^2^ compared to natives; ^3^ compared to high achievers; ^4^ compared to high SES adolescents.

## Data Availability

Publicly available datasets were analyzed in this study. This data can be found here upon free registration: INVALSI—Statistical Office https://invalsi-serviziostatistico.cineca.it (accessed on 7 June 2018).

## Appendix A

The Classmates Social Isolation Questionnaire (CSIQ). Peer acceptance: item 1, 3, 5, and 7. Peer friendship: item 2, 4, 6, and 8.

For each item, score ranges from 1 to 5, where 1 = no social contacts and 5 = social contacts with all the classmates.

- English Version

**Your classmates.** ***Put a cross in only one box for each question*.**	**None**	**Few**	**Some**	**Many**	**All**
1. How many of your classmates do you chat with?	□_1_	□_2_	□_3_	□_4_	□_5_
2. How many of your classmates do you meet outside school?	□_1_	□_2_	□_3_	□_4_	□_5_
3. How many of your classmates speak with you?	□_1_	□_2_	□_3_	□_4_	□_5_
4. How many of your classmates do you study with outside school?	□_1_	□_2_	□_3_	□_4_	□_5_
5. How many of your classmates do you talk with or exchange messages with on the mobile phone?	□_1_	□_2_	□_3_	□_4_	□_5_
6. How many of your classmates do you do activities with in your free time?	□_1_	□_2_	□_3_	□_4_	□_5_
7. How many of your classmates do you get on well with?	□_1_	□_2_	□_3_	□_4_	□_5_
8. How many of your classmates do you go out with, to have some fun?	□_1_	□_2_	□_3_	□_4_	□_5_

- Italian Version

**I tuoi compagni di classe.** ***Metti una crocetta su un solo quadratino per ogni riga*.**	**Nessuno**	**Pochi**	**Alcuni**	**Molti**	**Tutti**
1. Con quanti dei tuoi compagni di classe scambi due chiacchiere?	□_1_	□_2_	□_3_	□_4_	□_5_
2. Con quanti dei tuoi compagni di classe ti vedi fuori dalla scuola?	□_1_	□_2_	□_3_	□_4_	□_5_
3. Quanti dei tuoi compagni di classe parlano con te?	□_1_	□_2_	□_3_	□_4_	□_5_
4. Con quanti dei tuoi compagni di classe studi fuori dalla scuola?	□_1_	□_2_	□_3_	□_4_	□_5_
5. Con quanti compagni di classe ti telefoni o ti mandi messaggi al cellulare?	□_1_	□_2_	□_3_	□_4_	□_5_
6. Con quanti dei tuoi compagni di classe fai delle attività insieme nel tempo libero?	□_1_	□_2_	□_3_	□_4_	□_5_
7. Con quanti dei tuoi compagni di classe ti trovi bene?	□_1_	□_2_	□_3_	□_4_	□_5_
8. Con quanti dei tuoi compagni di classe ti capita di uscire per andare a divertirti?	□_1_	□_2_	□_3_	□_4_	□_5_
